# Fibulin‐5 null mice with decreased arterial compliance maintain normal systolic left ventricular function, but not diastolic function during maturation

**DOI:** 10.1002/phy2.257

**Published:** 2014-03-20

**Authors:** Victoria P. Le, Kellie V. Stoka, Hiromi Yanagisawa, Jessica E. Wagenseil

**Affiliations:** ^1^ Department of Biomedical Engineering Saint Louis University St. Louis Missouri; ^2^ Department of Mechanical Engineering and Materials Science Washington University St. Louis Missouri; ^3^ Department of Molecular Biology Southwestern Medical Center University of Texas Dallas Texas

**Keywords:** Arterial stiffness, echocardiography, elastin, hypertension, ultrasound

## Abstract

The large arteries serve as compliant vessels that store energy during systole and return it during diastole. This function is made possible by the elastic fibers in the arterial wall that are assembled during late embryonic and early postnatal development from various proteins, including fibulin‐5. Mice and humans with insufficient amounts of fibulin‐5 have reduced arterial compliance as adults. Reduced compliance of the large arteries is correlated with hypertension, reduced cardiac function, and an increased risk of death from cardiac and cardiovascular disease. The goal of this study was to quantify arterial compliance, blood pressure, and left ventricular (LV) function from early postnatal development to young adulthood in fibulin‐5 null (*Fbln5−/−*) mice to determine the effects of reduced arterial compliance during this critical period of elastic fiber assembly. We find that ascending aorta compliance is reduced as early as postnatal day (P) 7 and carotid artery compliance is reduced by P21 in *Fbln5−/−* mice. We did not find significant increases in systolic blood pressure by P60, but pulse pressures are increased by P21 in *Fbln5−/−* mice. LV systolic function, as measured by ejection fraction and fractional shortening, is unaffected in *Fbln5−/−* mice. However, LV diastolic function, as measured by tissue Doppler imaging, is compromised at all ages in *Fbln5−/−* mice. We propose that *Fbln5−/−* mice represent a suitable model for further studies to determine mechanistic relationships between arterial compliance and LV diastolic function.

## Introduction

Fibulin‐5 (FBLN5) is a component of the elastic fibers that allow the skin, lungs, and arteries to reversibly deform with physiologic loading (Yanagisawa and Davis 2010). Mutations in the FBLN5 protein lead to autosomal recessive cutis laxa type 1A (OMIM #219100), which is characterized by loose skin, emphysema, and vascular abnormalities (Loeys et al. 2002). Mice that do not express fibulin‐5 (*Fbln5−/−*) have loose skin, emphysema, and tortuous arteries with reduced compliance (Nakamura et al. 2002; Yanagisawa et al. 2002). Reduced compliance in *Fbln5−/−* carotid arteries is detectable as early as 3 weeks of age (Wan and Gleason 2013), but compliance has not been measured at earlier time points. Elastic fiber assembly occurs in late embryonic and early postnatal development and is essentially complete by 3 weeks of age (Wagenseil and Mecham 2009). Reduced compliance during this early time period may lead to adaptations in cardiovascular and cardiac function.

Reduced compliance (or increased stiffness) of the large arteries is associated with a higher risk of incident hypertension (Kaess et al. 2012), indicating that reduced compliance may be a cause of and not just a symptom of high blood pressure. This hypothesis is supported by studies in mice with elastin haploinsufficiency (*Eln+/−*) showing that reduced arterial compliance precedes increases in systolic blood pressure during postnatal development (Le et al. 2011). *Eln+/−* arteries have reduced unloaded diameters which may compound the effects of reduced arterial compliance on blood pressure and heart function. *Fbln5−/−* arteries do not have reduced unloaded diameters (Wan and Gleason 2013), hence *Fbln5−/−* mice are an ideal model to investigate the developmental time course of arterial compliance and blood pressure changes.

Reduced compliance of the large arteries is also associated with left ventricular (LV) diastolic dysfunction (Vriz et al. 2011). LV diastolic dysfunction is characterized by impaired filling, slow or delayed active relaxation, and/or decreased passive compliance (Gaasch and Zile 2004). Decreased compliance in both the large arteries and the LV, beyond that associated with aging and/or hypertension, is common in patients with heart failure with preserved ejection fraction (Kawaguchi et al. 2003). Almost half of all patients with heart failure have preserved ejection fraction and many of these patients have abnormal diastolic function. The reduced ventricular‐arterial compliance may exacerbate diastolic changes by compromising coupling between the heart and cardiovascular system (Borlaug and Kass 2008). *Eln+/−* mice show evidence of diastolic dysfunction, as measured by the ratio of peak mitral valve velocity of early rapid filling (E) to atrial filling (A), which inversely correlates with reduced arterial compliance (Le and Wagenseil 2012). However, more sophisticated measures of diastolic function, such as pulsed‐wave tissue Doppler imaging were not performed in *Eln+/−* mice.

In this study, we examine wild‐type (WT) and *Fbln5−/−* mice at three ages from early postnatal development to young adulthood to measure the temporal relationships between changes in arterial compliance, blood pressure, and LV function. We hypothesized that reduced arterial compliance due to disrupted elastic fibers in *Fbln5−/−* mice would precede increases in blood pressure and indications of LV diastolic dysfunction. LV diastolic dysfunction is difficult to diagnose and quantify in the clinic (Gaasch and Zile 2004), but arterial compliance measurements are becoming commonplace (Antonini‐Canterin et al. 2009), therefore correlations between the two may be useful for determining treatment programs to prevent heart failure.

## Materials and Methods

### Animals

Male and female *Fbln5−/−* (Budatha et al. 2011) and WT littermate mice at postnatal day (P) 7–8, 21–24, and 60–64 were used for all studies. The groups are named by the earliest age: P7, P21, and P60. Some mice were used at multiple time points, while others were sacrificed after examination for additional studies. In total, 53 P7, 47 P21, and 55 P60 mice were used for the study. All procedures were approved by the Institutional Animal Care and Use Committee.

### Blood pressure

Mice were weighed, anesthetized with 1.5% isoflurane and blood pressure measurements were performed using ultrasound‐guided LV puncture for P7 mice or a solid‐state catheter inserted into the ascending aorta through the carotid artery (1.2 Fr, Scisense is now owned by Transonic Systems Inc., Ithaca, NY) for older mice. The LV puncture method involves inserting a 25G needle attached to a fluid‐filled catheter (MLT 844, AD Instruments, Colorado Springs, CO) into the LV and recording the pressures with a PowerLab data acquisition system (AD Instruments). The LV puncture method does not have the temporal resolution to measure pulse pressures (PP; Le et al. 2012). P7 systolic blood pressure (SBP) was calculated as twice the mean LV pressure, assuming that LV diastolic blood pressure (DBP) was near zero. For P21 and P60 mice, arterial SBP, DBP, and PP were recorded.

### Echocardiography

Ultrasound examinations were performed with a Vevo 770 High‐Resolution Imaging System (VisualSonics, Toronto, ON, Canada) using the 707B (P7, P21) and 710 (P60) probes. Mice were weighed, anesthetized with 1.5% isoflurane, and their chest hair was removed with a chemical depilatory cream. Mice were secured to the imaging platform in the dorsal decubitus position and body temperature was maintained with a heat lamp (P7) or with a feedback controlled heating pad (P21, P60). Mice were examined using echocardiography to determine LV function and morphology, including LV mass/body weight (LVM/BW), LV inner diameter (LVID) at systole (s) and diastole (d), fractional shortening (FS), LV volume (LVV), stroke volume (SV), ejection fraction (EF), cardiac output/body weight (CO/BW), inner diameter of the ascending aorta (ASID), and carotid artery (CAID) at systole (s) and diastole (d), as described previously (Le and Wagenseil 2012). LVM was calculated using the Penn algorithm: LVM = 1.05*[(IVST + LVIDd + PWT)^3^ − (LVIDd)^3^], where IVST is the interventricular septum thickness, LVIDd is the LV inner diameter at end‐diastole, and PWT is the posterior wall thickness (Ghanem et al. 2006). Cardiac output was calculated by: CO = LV outflow tract velocity‐time integral × (aortic valve diameter/2)^2^ × 3.14 × heart rate, to avoid making assumptions about the LV geometry (Finsen et al. 2005). Transmitral flow velocity and tissue velocity at the mitral annulus on the LV lateral wall were measured using pulsed‐wave Doppler and tissue Doppler imaging, respectively. The ratio of peak LV filling velocity (E) to peak atrial contraction flow velocity (A), the deceleration time of early filling velocity (DT), the isovolumic relaxation time (IVRT), the ratio of peak early diastolic lateral LV wall velocity (E’) to the wall velocity during atrial contraction (A’) and E/E’ were calculated as indices of LV diastolic function. The peak systolic lateral LV wall velocity (S’) from tissue Doppler imaging was measured as an additional index of LV systolic function. All data were averaged from triplicate, blinded measurements by a single user (VPL) performed using VisualSonics software and following murine echocardiographic conventions (Pollick et al. 1995).

### Histology

To investigate histological changes related to the alterations in arterial compliance and LV diastolic function, mice were sacrificed by CO_2_ inhalation and the ascending aorta, carotid artery, and heart were removed and fixed in 10% formalin. The tissues were dehydrated in a graded series of ethanol, embedded in paraffin, sectioned, and stained with H&E to examine cell morphology, picrosirius red (PSR) to examine collagen content and Verhoeff Van Gieson (VVG) to examine elastic fibers.

### Protein quantification

Elastin and collagen protein amounts were measured in the LV to provide a quantitative measure of structural changes that may explain the functional alterations. The methods were modified from Long et al. (Long and Tranquillo 2003). Female mice were sacrificed by CO_2_ inhalation and the LV was removed, rinsed in saline, blotted dry, and weighed. The LV was digested in 0.1 mol/L NaOH to separate out insoluble elastin in the pellet from all other soluble proteins in the supernatant. The pellet and supernatant were hydrolyzed in 6 N HCl, and then dried in a speedvac system. A ninhydrin assay was used to quantify elastin in the pellet (Starcher 2001). Hydroxyproline, a major constituent of collagen, was measured through a reaction with Chloramine T (Stegemann and Stalder 1967). Hydroxyproline amounts were multiplied by 7.46 to obtain collagen amounts (Neuman and Logan 1950). Standards for elastin (Elastin Soluble, Elastin Products Company, Owensville, MO) and hydroxyproline (Trans‐4‐Hydroxy‐L_proline, Sigma–Aldrich, St. Louis, MO) were used to calibrate the colorimetric readings. All protein amounts were normalized to LV wet weight.

### Statistics

Data are presented as mean ± standard deviation. Each mouse was treated as a separate data point, as it was not possible to examine all mice at every age. The independent effects of age, sex, and genotype and interactions between the independent variables were determined using a general linear model (GLM) in SPSS (IBM, Armonk, NY). When genotype or age*genotype had a significant effect in the GLM, two‐tailed *t*‐tests with unequal variance between genotypes at each age were performed to further investigate differences between the genotypes. Linear regression analyses were performed to determine correlations between measures of arterial compliance and cardiac function. *P* < 0.05 was considered significant.

## Results

### General linear model

The statistical results for the GLM are shown in Table 1. All parameters except PP, CO/BW, A, DT, IVRT, S’, E/E’, and LV elastin amounts are significantly affected by age. BW and LV volume are the only measurements significantly affected by sex, so males and females are combined for all other measurements. DP, PP, BW, LVM, LVM/BW, ascending aorta (AS) and carotid artery (CA) dimensions, measures of diastolic function (A, E/A, E’, A’, E’/A’, E/E’), and LV collagen amounts are all significantly affected by genotype or show an interaction between age and genotype.

**Table 1:** Results of the GLM for each variable including independent effects of age, sex, and genotype (GT) and interactions between the independent variables. Significant values (*P* < 0.05) and good fits to the GLM (*R* ^2^ > 0.7) are shown in bold. Two‐tailed *t*‐tests with unequal variance were performed to compare genotypes at each age when genotype or genotype*age had a significant effect in the GLM. Definitions for each variable are given in the footnote below

Variable	Age	Sex	GT	Age*Sex	GT*Sex	GT*Age	GT*Age*Sex	*R* ^2^
SP	**0.000**	0.147	0.154	**0.000**	**0.001**	**0.007**	**0.000**	**0.872**
DP	**0.000**	0.215	**0.000**	**0.024**	**0.003**	0.255	**0.001**	0.652
PP	0.364	0.102	**0.000**	0.000	0.188	**0.038**	0.340	0.627
BW	**0.000**	**0.000**	**0.003**	**0.000**	0.767	**0.003**	0.484	**0.967**
LVM	**0.000**	0.378	**0.003**	**0.071**	0.436	0.170	0.908	**0.889**
LVM/BW	**0.000**	0.130	**0.001**	0.313	0.619	0.181	0.985	0.397
LVIDdias	**0.000**	0.938	0.547	**0.035**	0.742	0.599	0.692	**0.941**
LVIDsys	**0.000**	0.472	0.710	0.293	0.453	0.668	0.830	**0.838**
FS	**0.000**	0.155	0.430	0.414	0.205	0.469	0.512	0.480
LVVd	**0.000**	**0.032**	0.392	**0.011**	0.688	0.384	0.352	**0.924**
LVVs	**0.000**	**0.042**	0.219	0.139	0.951	0.389	0.984	**0.825**
SV	**0.000**	0.473	0.763	0.138	0.627	0.847	0.220	**0.831**
EF	**0.001**	0.662	0.566	0.330	0.809	0.284	0.555	0.301
ASIDdias	**0.000**	0.669	**0.014**	0.532	0.269	0.257	0.429	**0.857**
ASIDsys	**0.000**	0.268	**0.000**	0.596	0.691	**0.003**	0.960	**0.883**
ASID%inc	**0.005**	0.254	**0.000**	0.349	0.352	**0.004**	**0.036**	0.590
CAIDdias	**0.000**	0.237	**0.007**	**0.028**	0.309	0.137	0.403	**0.731**
CAIDsys	**0.000**	0.467	**0.000**	0.370	0.055	**0.001**	0.760	**0.933**
CAID%inc	**0.000**	0.133	**0.000**	0.214	0.264	0.084	0.825	0.502
AVDs	**0.000**	0.448	0.348	0.811	**0.043**	0.303	0.066	**0.861**
HR	**0.000**	0.945	0.175	0.809	0.203	0.054	0.919	0.444
CO	**0.000**	0.248	0.938	0.187	0.165	0.896	0.380	0.596
CO/BW	0.642	0.592	0.651	0.186	**0.044**	0.533	0.712	0.130
E	**0.002**	0.487	0.654	0.348	0.731	0.306	0.460	0.209
A	0.196	0.445	**0.021**	0.445	0.544	**0.009**	0.141	0.270
E/A	**0.000**	0.997	**0.001**	0.729	0.776	0.157	0.657	0.325
DT	0.087	0.131	0.180	0.474	**0.044**	0.066	0.034	0.275
IVRT	0.612	0.853	0.090	0.548	0.939	0.497	0.819	0.096
E’	**0.004**	0.053	**0.000**	0.817	0.201	0.974	0.572	0.347
S’	0.062	0.117	0.524	**0.012**	0.174	0.312	0.697	0.233
A’	**0.020**	0.213	**0.001**	0.237	0.200	**0.025**	0.902	0.332
E’/A’	**0.000**	0.203	**0.000**	0.338	**0.022**	**0.008**	0.648	0.627
E/E’	0.398	0.142	**0.000**	0.252	0.711	0.052	0.170	0.317
Collagen	**0.001**	N/A	0.326	N/A	N/A	**0.043**	N/A	0.483
Elastin	0.585	N/A	0.585	N/A	N/A	0.899	N/A	0.127

GLM, general linear model; SP, systolic pressure; DP, diastolic pressure; PP, pulse pressure; BW, body weight; LVM, left ventricular (LV) mass; LVM/BW, LV mass normalized to body weight; LVIDdias, LV inner diameter at diastole; LVIDsys, LV inner diameter at systole; FS, fractional shortening; LVVd, LV volume at diastole; LVVs, LV volume at systole; SV, stroke volume; EF, ejection fraction; ASIDdias, ascending aortic inner diameter at diastole; ASIDsys, ascending aortic inner diameter at systole; ASID%inc, percent increase of the ascending aorta from diastole to systole; CAIDdias, carotid artery inner diameter at diastole; CAIDsys, carotid artery inner diameter at systole; CAID%inc, percent increase of the carotid artery from diastole to systole; AVDs, aortic valve diameter at systole; HR, heart rate; CO, cardiac output; CO/BW, cardiac output normalized to body weight; E, peak velocity of the early filling wave through the mitral valve; A, peak velocity of the atrial filling wave through the mitral valve; E/A, ratio of the early to atrial filling wave velocities; DT, deceleration time of the early filling velocity; IVRT, isovolumic relaxation time; E’, peak velocity of the LV lateral wall during early filling; S’, peak velocity of the LV lateral wall during systole; A’, peak velocity of the LV lateral wall during atrial filling; E’/A’, ratio of the LV wall velocities during early and atrial filling; E/E’, ratio of the wave velocity to the LV wall velocity during early filling; Collagen, normalized LV collagen content; Elastin, normalized LV elastin content.

### Blood pressure

**Figure 1:**
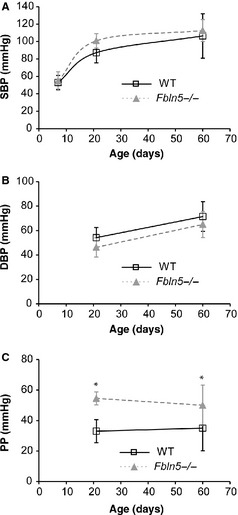
There are no differences in systolic blood pressure (SBP) (A) or diastolic blood pressure (DBP) (B) between genotypes. Pulse pressure (PP) (C) is elevated in *Fbln5−/−* mice at P21 and P60. Pressures were measured by left ventricular (LV) puncture at P7 (Le et al. 2012), which cannot measure arterial PP, and insertion of a solid‐state catheter in the ascending aorta at P21 and P60. *P < 0.05 between genotypes. *n* = 11–21 per group.

There are no significant differences between genotypes for SBP (Fig. 1A) or DBP (Fig. 1B), but PP is 65% (*P* < 0.0005) and 43% (*P* = 0.01) higher in *Fbln5−/−* mice compared to WT at P21 and P60, respectively (Fig. 1C). PP cannot be measured in P7 mice using the LV puncture method (Le et al. 2012). The changes in SBP with age in WT mice are similar to previous studies (Le et al. 2011), but unlike previous studies using *Eln+/−* mice with reduced arterial compliance, we did not find significantly increased SBP in *Fbln5−/−* mice by P60.

### Body weight (BW) and left ventricle mass (LVM)

**Figure 2:**
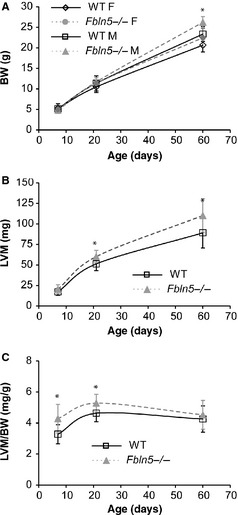
Body weight (BW) (A) increases with age, is affected by sex, and is higher in *Fbln5−/−* mice at P60. Left ventricle mass (LVM) (B) is increased in *Fbln5−/−* mice at P21 and P60. Normalized LVM mass (LVM/BW) (C), an indication of left ventricular (LV) hypertrophy, is increased in *Fbln5−/−* mice at P7 and P21. *P < 0.05 between genotypes. *n* = 5–15 per group (A). *n* = 14–23 per group (B, C).

P60 *Fbln5−/−* mice weigh 9–12% (*P* = 0.02–0.03) more than WT (Fig. 2A). Wan et al. (Wan and Gleason 2013) found a slight increase in body weight at this age that was not significant. LVM is about 20% higher in *Fbln5−/−* mice at P21 (*P* = 0.01) and P60 (*P* = 0.003; Fig. 2B). Normalized LV mass in *Fbln5−/−* mice is 31% higher at P7 (*P* = 0.005), 14% higher at P21 (*P* = 0.01), and not significantly different from WT at P60 (Fig. 2C). It appears that LV hypertrophy occurs early in *Fbln5−/−* postnatal development, but then the mice are able to adapt and bring LVM/BW to WT values in young adulthood. The changes in LV mass with age are similar to previous studies (Ghanem et al. 2006).

### Ejection fraction (EF) and stroke volume (SV)

**Figure 3:**
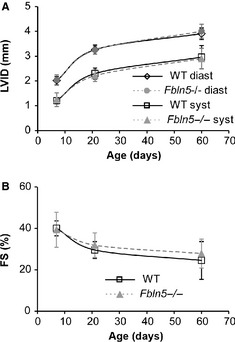
Left ventricular inner diameters (LVID) (A) at diastole and systole increase with age, but are not affected by genotype (A). Fractional shortening (FS) (B), a measure of systolic function, is not affected by genotype. *n* = 11–20 per group.

**Figure 4:**
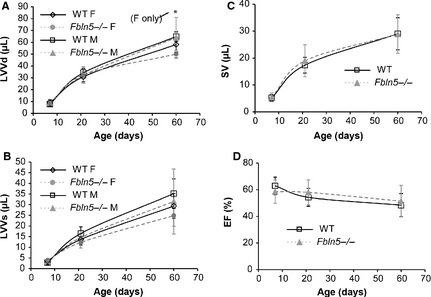
Left ventricular (LV) volumes at end‐diastole (LVVd) (A) and end systole (B) increase with age, are affected by sex, and in general are not different between genotypes. Stroke volume (SV) (C) increases with age and is not affected by sex or genotype. Ejection fraction (EF) (D), a measure of systolic function, decreases slightly with age and is not affected by sex or genotype. *P < 0.05 between genotypes. *n* = 3–12 per group (A, B). *n* = 9–17 per group (C, D).

There are no significant differences between genotypes for LVID (Fig. 3A) or FS (Fig. 3B). LVV at diastole is 14% lower in P60 female *Fbln5−/−* mice (*P* = 0.003; Fig. 4A), but there are no significant differences between genotypes for any age or sex for LVV at systole (Fig. 4B), SV (Fig. 4C) or EF (Fig. 4D), indicating that there is no dilation of the LV chamber and LV systolic function is normal in *Fbln5−/−* mice from P7 to P60. The changes in LV size and systolic function with age are similar to previous studies (Le et al. 2012).

### Cardiac output (CO)

**Figure 5:**
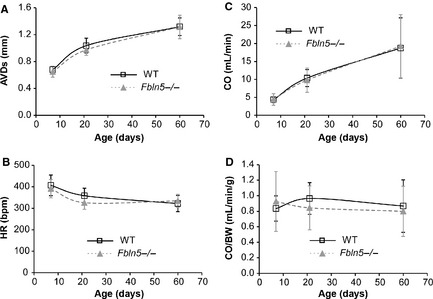
The end‐systolic diameter of the aortic valve orifice (AVDs) (A) increases with age and is not affected by genotype. Heart rate (HR) (B) decreases with age and is not affected by genotype. Cardiac output (CO) (C) increases with age and is not affected by genotype. Normalized cardiac output (CO/BW) (D) is not affected by age or genotype. *P < 0.05 between genotypes. *n* = 12–22 per group.

There are no significant differences between genotypes for the size of the aortic valve orifice at systole (AVDs; Fig. 5A), heart rate (HR; Fig. 5B), CO (Fig. 5C) or CO/BW (Fig. 5D), indicating that perfusion of distal tissues is normal in *Fbln5−/−* mice from P7 to P60. Cardiac output significantly depends on age, but CO/BW is independent of age (Table 1). In *Eln+/−* mice, the aortic valve orifice is smaller than WT at P60, reducing CO by 20% (Le et al. 2012). Reduced CO may impact downstream perfusion, complicating analyses of relationships between arterial compliance and heart function in *Eln+/−* mice. *Fbln5−/−* mice offer a simpler model for determining the direct effects of changes in arterial compliance on heart function.

### Arterial size and compliance

**Figure 6:**
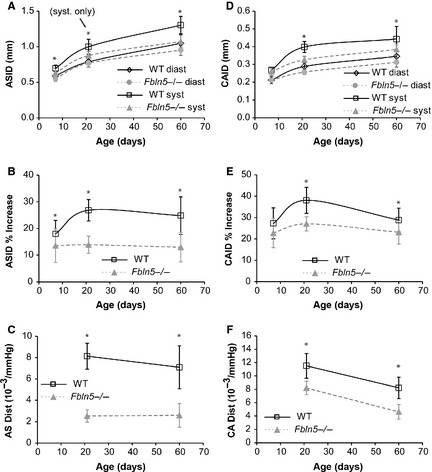
Ascending aorta inner diameter (ASID) (A) increases with age and is affected by genotype. The percent increase of the ASID (B) is reduced in *Fbln5−/−* mice at all ages. The distensibility of the ascending aorta (C) is reduced in *Fbln5−/−* mice at P21 and P60. Distensibility cannot be calculated at P7, because we cannot measure arterial pulse pressures at this age. Carotid artery inner diameter (CAID) (D) increases with age and is affected by genotype. The percent increase of the CAID (E) is reduced in *Fbln5−/−* mice at P21 and P60. The distensibility of the carotid artery (F) is reduced in *Fbln5−/−* mice at P21 and P60. *P < 0.05 between genotypes. *n* = 10–22 per group.

Ascending aorta inner diameter (ASID) at systole is 10–20% (*P* < 0.0005–0.01) smaller in *Fbln5−/−* mice compared to WT at all ages (Fig. 6A). ASID at diastole is 6% (*P* = 0.03) and 9% (*P* = 0.008) smaller in *Fbln5−/−* mice compared to WT at P7 and P60, respectively. The percent increase in ASID from diastole to systole is 26–48% (*P* < 0.0005–0.05) smaller in *Fbln5−/−* mice compared to WT at all ages (Fig. 6B). Normalized diameter compliance, or distensibility = (D_s_−D_d_)/(D_d_*PP); (Cavalcante et al. 2011), was calculated using the average PP for each genotype at P21 and P60 because we did not perform blood pressure measurements simultaneously with ultrasound imaging and PP measurements were not possible at P7. Distensibility is 69% (*P* < 0.0005) and 63% (*P* < 0.0005) smaller in *Fbln5−/−* aorta compared to WT at P21 and P60, respectively (Fig. 6C). Carotid artery inner diameter at systole is 18% (*P* < 0.0005) and 13% (*P* = 0.004) smaller than WT at P21 and P60, respectively (Fig 6D). Carotid artery inner diameter at diastole is 11% (*P* < 0.0005) and 9% (*P* < 0.05) smaller than WT at P21 and P60, respectively. The percent increase in CAID from diastole to systole is 29% (*P* < 0.0005) and 20% (*P* = 0.003) smaller in *Fbln5−/−* mice compared to WT at P21 and P60, respectively (Fig. 6E). Distensibility is 29% (*P* < 0.0005) and 44% (*P* < 0.0005) smaller in *Fbln5−/−* carotid artery compared to WT at P21 and P60, respectively (Fig. 6F). The changes in arterial size and percent change in diameter with age in WT mice are similar to previous results. *Fbln5−/−* mice have similar decreases in the percent change in diameter as *Eln+/−* mice compared to WT at most ages (Le and Wagenseil 2012).

### Diastolic function

**Figure 7:**
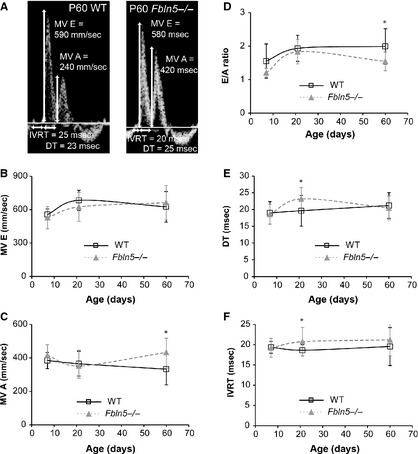
Representative Doppler images (A) with mitral valve (MV) early (E) and atrial (A) peak velocity values, and measurements of isovolumic relaxation times (IVRT) and deceleration times (DT) in P60 wild‐type (WT) and *Fbln5−/−* mice. MV E (B) is affected by age, but not genotype. MV A (C) is affected by genotype, but not age. The E/A ratio (D), a measure of diastolic function, is affected by age and genotype. DT (E) and IVRT (F) are increased in *Fbln5−/−*mice at P21. *P < 0.05 between genotypes. *n* = 10–22 per group.

Representative measurements for the mitral valve velocities and time intervals are shown in Figure 7A. There are no significant differences between genotypes for MV E wave velocities (Fig. 7B). MV A wave velocity is 30% (*P* = 0.001) higher in *Fbln5−/−* mice compared to WT at P60 (Fig. 7C). The E/A ratio is reduced 22% (*P* = 0.003) in *Fbln5−/−* mice compared to WT at P60 (Fig. 7D). DT is increased 18% (*P* = 0.03; Fig. 7E) and IVRT is increased 11% (*P* = 0.04; Fig. 7F) in *Fbln5−/−* mice at P21. Increased A wave velocity, DT, and IVRT and reduced E/A ratio are indicative of LV diastolic dysfunction (Shapiro and Gibson 1988). The changes in mitral valve velocities and time intervals with age in WT mice are similar to previous results. *Eln+/−* mice showed evidence of LV diastolic dysfunction over the same age range (Le and Wagenseil 2012).

**Figure 8:**
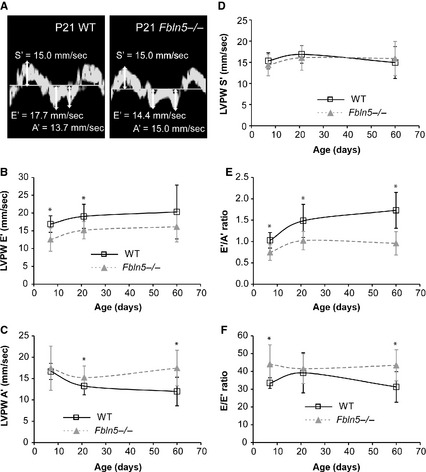
Representative tissue Doppler images (A) with left ventricular (LV) posterior wall (PW) velocities during systole (S’) and during early (E’) and atrial (A’) filling in P21 wild‐type (WT) and *Fbln5−/−* mice. LVPW E’ (B) velocity is decreased in *Fbln5−/−* mice at P7 and 21. LVPW A’ (C) velocity is increased in *Fbln5−/−* mice at P21 and P60. LVPW S’ (D) velocity is similar between genotypes at all ages. The E’/A’ (E) and E/E’ (F) ratios, which are indices of diastolic function, are different in *Fbln5−/−* mice compared to WT at most ages. *P < 0.05 between genotypes. *n* = 10–16 per group.

Representative measurements for the LV tissue velocity are shown in Figure 8A. The E’ velocity is reduced 26% (*P* = 0.001) and 20% (*P* = 0.001) in *Fbln5−/−* mice at P7 and P21, respectively (Fig. 8B), indicating impaired LV relaxation. The A’ velocity is increased 15% (*P* = 0.03) and 46% (*P* = 0.001) in *Fbln5−/−* mice at P21 and P60, respectively (Fig. 8C). The S’ velocity is similar in WT and *Fbln5−/−* mice, indicating normal systolic function (Fig. 8D). The E’/A’ ratio is reduced 28–45% at all ages (*P* < 0.0005 for all) in *Fbln5−/−* mice compared to WT (Fig. 8E). The E/E’ ratio is increased 32% (*P* = 0.005) and 39% (*P* = 0.002) in *Fbln5−/−* mice at P7 and P60 (Fig. 8F), indicating increased LV filling pressures (Nagueh 2009). Reduced E’/A’ and increased E/E’ show the best correlation (compared to E/A, DT, and IVRT) with invasive measures of diastolic dysfunction in humans with heart failure and normal ejection fraction (Kasner et al. 2007).

### Linear regression analyses

**Figure 9:**
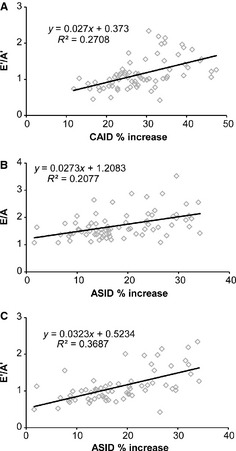
There are weak correlations between measures of carotid artery (A) or ascending aorta compliance (B and C) (as measured by percent increase of the inner diameter) and diastolic function (as measured by ratios of pulsed wave, E/A, and tissue Doppler, E’/A’, filling velocities) in maturing mice.

To further investigate the relationship between arterial compliance and cardiac function, we determined correlations between the percent change in arterial diameter (as a measure of arterial compliance) and LVM/BW (as a measure of cardiac hypertrophy), FS, EF, and S’ (as measures of systolic function), and E/A, E’/A’, and E/E’ (as measures of diastolic function). There are no correlations between ASID or CAID% increase and LVM/BW, FS, EF, S’, or E/E’ (*R* ^2^ < 0.1). There are weak correlations (0.2 < *R* ^2^ < 0.5) between ASID or CAID% increase and E/A or E’/A’ (Fig. 9). The strongest correlation is between ASID% increase and E’/A’, indicating that reduced aortic compliance correlates with diastolic dysfunction in maturing mice.

### Histology

**Figure 10:**
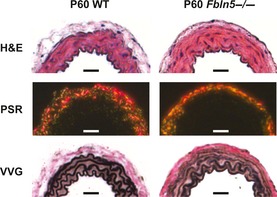
Representative histology sections of P60 carotid arteries. H&E staining and picrosirius red staining (PSR) observed under polarized light show no obvious differences between genotypes. Verhoeff Van Gieson (VVG) staining shows less intense staining of the elastic lamellae in *Fbln5−/−* arteries, especially in the outer layers. Scale bars = 30 *μ*m. Histology sections from six different mice were examined for each group.

Histological images of the ascending aorta and carotid artery show defects in the elastic fibers of *Fbln5−/−* mice at all ages, with no obvious differences in the cell morphology or collagen arrangement. Representative images of P60 carotid arteries are shown in Figure 10. The elastic fiber staining is less intense in *Fbln5−/−* carotid arteries, especially in the outer layers. The images are consistent with previously published data on the elastic fibers in *Fbln5−/−* mice. The reduced staining intensity is most likely due to fragmentation of the elastic fibers than can be observed with electron microscopy (Nakamura et al. 2002; Yanagisawa et al. 2002).

**Figure 11:**
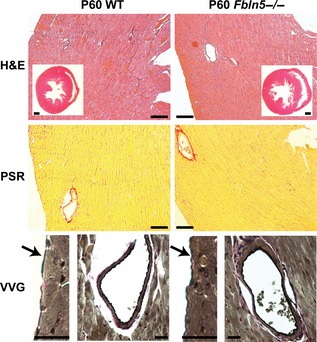
Representative histology sections of P60 mouse hearts. H&E staining of the entire heart and of the left ventricular (LV) free wall show no differences between genotypes. Picrosirius red (PSR) staining of the LV free wall shows no differences in collagen density between genotypes. Verhoeff Van Gieson (VVG) staining of the thin pericardial layer (arrow) shows intact elastic fibers in both genotypes. VVG staining of coronary vessels shows an intact internal elastic lamina in both genotypes and no elastin staining between cardiomyocyte layers. H&E inset scale bars = 500 *μ*m, H&E and PSR scale bars = 100 *μ*m, VVG scale bars = 20 *μ*m. Histology sections from six different mice were examined for each group.

Histological images of the LV did not present any obvious explanations for the decreased diastolic function in *Fbln5−/−* mice (Fig. 11). H&E staining shows similar LV size, and cardiomyocyte size and density between genotypes at each age. Picrosirius red staining exhibits similar collagen fiber density between genotypes, with faint collagen staining between cardiomyocyte layers, and bright collagen staining in the adventitia of coronary vessels. VVG staining demonstrates a thin layer of elastin and collagen in the pericardium, an absence of elastin in the myocardium, and a prominent internal elastic lamina in the coronary vessels of both genotypes.

### LV protein quantification

**Figure 12:**
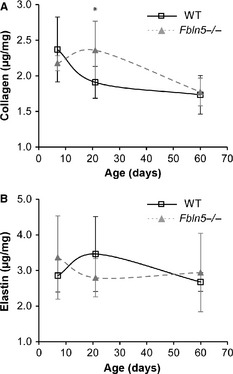
Normalized collagen (A) and elastin (B) content in the left ventricle. Collagen content is affected by age, but elastin content is not. There are increased collagen amounts in P21 *Fbln5−/−* aorta, with no differences at other ages. There are no differences in the elastin amounts between groups. *n* = 6 per group.

Normalized collagen content decreases with age, whereas normalized elastin content remains constant with age (Fig. 12). There is 24% more collagen in P21 *Fbln5−/−* LV than WT (*P* = 0.046; Fig. 12A), with no significant differences between genotypes at other ages. The observed LV diastolic dysfunction may be caused by collagen deposition that increases the LV passive stiffness in *Fbln5−/−* mice. There are no differences in LV elastin content between genotypes at any age (Fig. 12B). The measured collagen content is similar to previous studies on adult rat LV, whereas the elastin content is about an order of magnitude higher than previous studies (Ruzicka et al. 1994). Differences in elastin content may be due to species differences, variations in the quantification protocol, or the standards used for calibrating the colorimetric readings. Regardless of differences in the absolute protein amounts, relative comparisons between genotypes and ages can still be made.

## Discussion

The large arteries serve as “windkessels” that distend during systole and relax during diastole, pushing blood to distal vessels, and dampening the pulse wave. This behavior reduces LV afterload and improves coronary blood flow and LV relaxation (Belz 1995). LV afterload is determined by the overall arterial impedance, which includes the resistance, compliance, and wave reflection of the cardiovascular system (O'Rourke 1991). In an ideal case, the large arteries are highly compliant and late wave reflection augments pressure in diastole. In disease and aging, arterial compliance is reduced and amplification of the forward wave and/or early reflections of the reverse wave augment SBP, increasing PP and LV afterload and decreasing coronary blood flow. We show that a mouse model of cutis laxa (*Fbln5−/−*) with disorganized elastic fibers in the large arteries has altered windkessel behavior as early as P7 and investigate the resulting effects on blood pressure and LV function during maturation.

### Elastic fibers, arterial compliance, and maturation

Elastic fiber assembly is a complex process that is, regulated spatially and temporally. Spatially, smooth muscle cells in the arterial wall secrete soluble tropoelastin which forms aggregates on the cell surface (Kozel et al. 2006). Fibulin‐5 preferentially binds tropoelastin (Zheng et al. 2007) and may help organize the coacervation of tropoelastin molecules for crosslinking by lysyl oxidase and interaction with microfibrils (Yanagisawa and Davis 2010). When the spatial pattern is disturbed by the absence of *Fbln5*, the arterial elastic fibers are disorganized and fragmented. Fragmentation can be observed by weak staining of the elastic fibers at the level of light microscopy or discrete elastin aggregates, instead of continuous laminae, at the level of electron microscopy. This leads to reduced arterial compliance and high systolic and pulse pressures in adult *Fbln5−/−* mice (Nakamura et al. 2002; Yanagisawa et al. 2002). Temporally, expression of the elastin gene is highest between P4 to P21 in developing mouse aorta (Kelleher et al. 2004). In fact, most extracellular matrix (ECM) proteins begin expression late in embryonic development, peak between P7 to P21 and then return to baseline expression levels in the adult mouse (Larsson et al. 2008). Our results show that the percent change in diameter is reduced in *Fbln5−/−* aorta as early as P7, when the lack of *Fbln5* expression may have a large impact on elastic fiber assembly. During maturation, the percent change in diameter for WT and *Fbln5−/−* arteries peaks around P21 and then declines, mimicking the ECM gene expression pattern. Similar timing for the percent change in diameter was observed in WT and *Eln+/−* mouse arteries (Le and Wagenseil 2012). The change in compliance of the arteries may reflect the addition of ECM proteins during this critical maturation period.

### Arterial compliance and hypertension

In a longitudinal study of 1759 individuals over 7 years, decreased arterial compliance (as measured by pulse wave velocity) was associated with a higher risk of incident hypertension, while initial blood pressure was not associated with a risk of further decreases in arterial compliance (Kaess et al. 2012). This confirms previous observations that reduced arterial compliance is an independent predictor, and may be a partial cause of, incident hypertension (Dernellis and Panaretou 2005; Takase et al. 2011). With animal studies, we can better dissect the temporal relationship between arterial compliance and hypertension. We show here that the percent change in diameter between diastole and systole is reduced as early as P7 in the aorta and P21 in the carotid artery. Due to limitations with pressure measurements in young mice (Le et al. 2012), we cannot calculate compliance at P7, but the normalized aortic and carotid compliance (or distensibility) are reduced at P21 in *Fbln5−/−* mice. Based on the percent change in diameter measurements, the distensibility of *Fbln5−/−* aorta would be reduced compared to WT as early as P7 if the pulse pressures are equal. A higher pulse pressure in P7 *Fbln5−/−* mice, as observed at P21 and P60, would exaggerate the distensibility differences even more. We did not find significant increases in SBP in *Fbln5−/−* mice by P60. Previous reports have shown increased SBP in *Fbln5−/−* mice with a different genetic background by P90 (Yanagisawa et al. 2002), supporting the assertion that reduced arterial compliance precedes changes in SBP.

Although SBP is not increased at the ages we studied, PP is significantly higher at P21 and P60 in *Fbln5−/−* mice. Increased PP causes remodeling of the microcirculation that increases the resistance to flow (James et al. 1995; Baumbach 1996) and may cause endothelial dysfunction (Ryan et al. 1995). PP is a better predictor of congestive heart failure in elderly individuals than SBP (Chae et al. 1999) and is the best predictor of coronary heart disease in middle aged individuals (Franklin et al. 1999). Because of the high PP at an early age, *Fbln5−/−* mice may be a useful model to further investigate relationships between PP and cardiovascular and cardiac disease.

### Arterial compliance and LV function

Decreased arterial compliance directly correlates with increased LV mass (Isnard et al. 1989; Toprak et al. 2009; Cernes et al. 2010), decreased systolic function (Chow et al. 2007; Fernandes et al. 2008; Noguchi et al. 2011) and diastolic dysfunction (Borlaug et al. 2007; Mizuguchi et al. 2007; el Ibrahim et al. 2011) in humans. Recent studies in mice have confirmed correlations between arterial compliance and LV function in interleukin 10 knockout mice (Sikka et al. 2013), diabetic mice (Reil et al. 2013), and *Eln+/−* mice (Le and Wagenseil 2012). *Eln+/−* and *Fbln5−/−* mice both show no decrease in LV systolic function, but indications of impaired LV diastolic function early in maturation. In senescence‐accelerated mice, diastolic dysfunction is present without any changes in systolic function (Reed et al. 2011). Diastolic dysfunction may be caused by changes in the cardiomyocytes or the ECM proteins, especially collagen, in the LV wall (Gaasch and Zile 2004).

Although we did not detect differences in the cardiomyocytes or ECM proteins in the LV wall by light microscopy, protein quantification shows that collagen amounts are increased in P21 *Fbln5−/−* mice compared to WT. Increased collagen deposition could increase the passive stiffness of the *Fbln5−/−* LV and lead to diastolic dysfunction. We believe that increased LV collagen deposition is a direct response to alterations in the mechanical coupling between the LV and the cardiovascular system caused by decreased arterial compliance in *Fbln5−/−* mice. Variations in the collagen type (Mukherjee and Sen 1990), glycation (Herrmann et al. 2003), and crosslinking (Badenhorst et al. 2003) can also change the LV passive stiffness and affect diastolic function. Additional studies on the physical changes in the LV of *Fbln5−/−*mice are needed to further investigate the mechanisms underlying the observed diastolic dysfunction.

There were no differences in LV elastin amounts between genotypes, but it is possible that disorganized elastic fibers in the LV contribute directly to diastolic dysfunction, rather than being a downstream effect of reduced arterial compliance. Consistent with reports in porcine LV (Sato et al. 1983), we found elastin in the pericardium and in association with coronary vessels, but not in between cardiomyocyte layers. In porcine LV, disrupting the pericardium changes the passive mechanical behavior and residual stress, implying that elastin plays a significant role in diastolic function (Jobsis et al. 2007). We did not see evidence of disrupted elastic fibers in the *Fbln5−/−* pericardium, supporting our hypothesis that LV diastolic dysfunction is directly related to reduced arterial compliance in these mice. The elastic fibers in the coronary vessels also appeared intact, which is consistent with reports in the large arteries that the internal elastic lamina is less affected by *Fbln5* deficiency than the elastic laminae in the medial layer (Chapman et al. 2010). There are distinct layers of elastic fibers in the atrialis of the mitral valve and they are disrupted in individuals with mitral valve prolapse syndrome (Nasuti et al. 2004). Changes in the geometry or mechanics of the mitral valve could have a direct result on the LV filling velocities that we observed, hence mitral valve structure and function is an important area for future investigation in *Fbln5−/−* mice.

Diastolic dysfunction precedes diastolic heart failure, which is characterized by heart failure with preserved LV ejection fraction and abnormal diastolic indices. Approximately 30% of individuals with heart failure exhibit diastolic heart failure, especially in patients with hypertension and in the elderly (Gaasch and Zile 2004). There are arguments that diastolic heart failure and systolic heart failure are not separate diseases, but different phenotypes of the same disease (De Keulenaer and Brutsaert 2007). In individuals with diastolic heart failure, tissue Doppler imaging identifies systolic abnormalities not measurable with global ejection fraction measurements (Yu et al. 2002). Additionally, a significant number of diastolic heart failure patients may progress to systolic heart failure (Cahill et al. 2006). *Fbln5−/−*mice offer a model of diastolic dysfunction for investigating heart failure progression and possible pharmaceutical treatments.

## Limitations

Mouse models are useful for examining the effects of specific gene targets on cardiovascular and cardiac function. Care must be taken in interpreting these results, however, because genetic, biochemical, structural, and physiological differences exist that may preclude translation of results obtained in mice to humans. The small size and high heart rate of mice make functional measurements challenging. Advances in ultrasound technology have facilitated these measurements in mice, especially for diastolic function, which is less well defined than systolic function. Other techniques, such as MRI, have better spatial and temporal resolution, but are costly and have more limited access. We used *Fbln5−/−* mice to investigate how reduced arterial compliance affects blood pressure and LV function, but size and temporal variations between mice and humans may alter the timing of pulse waves from the heart so that the consequences of reduced arterial compliance are different in each organism. Our measurements were performed in anesthetized mice which will influence blood pressure, heart rate, and LV function. However, isoflurane has minimal depression of cardiac function and provides stable hemodynamic conditions, compared to other anesthetics (Roth et al. 2002).

Other factors can cause changes in the mitral filling velocities and LV tissue velocities besides diastolic dysfunction. These include constrictive pericarditis, mitral stenosis, and pulmonary hypertension (Nagueh et al. 2009). Additional studies must be performed to examine modifications to the mitral valves, left atrium, and right ventricle that may cause changes in the filling and tissue velocities. Invasive LV measures, such as the pressure gradient and end‐diastolic pressure, in future studies would add support to our observations of LV diastolic dysfunction. We performed measurements in P7–P60 mice to focus on early maturation when ECM protein expression is high, but additional measurements must be carried out in older mice to follow LV function with aging.

## Conclusions

*Fbln5−/−* mice have reduced arterial compliance as early as P7, as measured by the percent change in diameter of the ascending aorta. *Fbln5−/−* mice have increased PP at P21, which is the earliest age that we can measure PP using our techniques. *Fbln5−/−* mice have normal systolic function, as measured by FS and EF, but have indications of diastolic dysfunction, as measured by tissue Doppler imaging, as early as P7. We propose that *Fbln5−/−* mice represent a good model for further investigations on the mechanisms and temporal relationships between arterial compliance and diastolic dysfunction.

## Conflict of Interest

None declared.
